# Management of Occupational Risk Prevention of Nanomaterials Manufactured in Construction Sites in the EU

**DOI:** 10.3390/ijerph17249211

**Published:** 2020-12-09

**Authors:** Mónica López-Alonso, Beatriz Díaz-Soler, María Martínez-Rojas, Carlos Fito-López, María Dolores Martínez-Aires

**Affiliations:** 1 Department of Construction Engineering and Projects, University of Granada, 18010 Granada, Spain; mlopeza@ugr.es; 2 Department of Building Construction, University of Granada, 18010 Granada, Spain; bmdiazsoler@gmail.com; 3 Department of Business Administration and Economics, University of Málaga, 29016 Málaga, Spain; mmrojas@uma.es; 4 ITENE, Technological Institute of Packaging, Transport and Logistics, 46980 Paterna, Spain; carlos.fito@itene.com

**Keywords:** nanosafety, engineered nanomaterials, construction site, occupational risk prevention management, safety protocol

## Abstract

Currently, nanotechnology plays a key role for technological innovation, including the construction sector. An exponential increase is expected in its application, although this has been hampered by the current degree of uncertainty regarding the potential effects of nanomaterials on both human health and the environment. The accidents, illnesses, and disease related to the use of nanoproducts in the construction sector are difficult to identify. For this purpose, this work analyzes in depth the products included in recognized inventories and the safety data sheets of these construction products. Based on this analysis, a review of the recommendations on the use of manufactured nanomaterials at construction sites is performed. Finally, a protocol is proposed with the aim of it serving as a tool for technicians in decision-making management at construction sites related to the use of manufactured nanomaterials. This proposed protocol should be an adaptive and flexible tool while the manufactured nanomaterials-based work continues to be considered as an “emerging risk,” despite the expectation that the protocol will be useful for the development of new laws and recommendations for occupational risk prevention management.

## 1. Introduction

In the last decade, nanotechnology has become one of the research areas with the greatest technological and scientific growth; it is one of the most important technologies in the development of new products. Additionally, it has been defined as one of the main technologies to address the social challenges within the European Framework Program for Research and Innovation of the EU HORIZON 2020.

**Figure 1:**
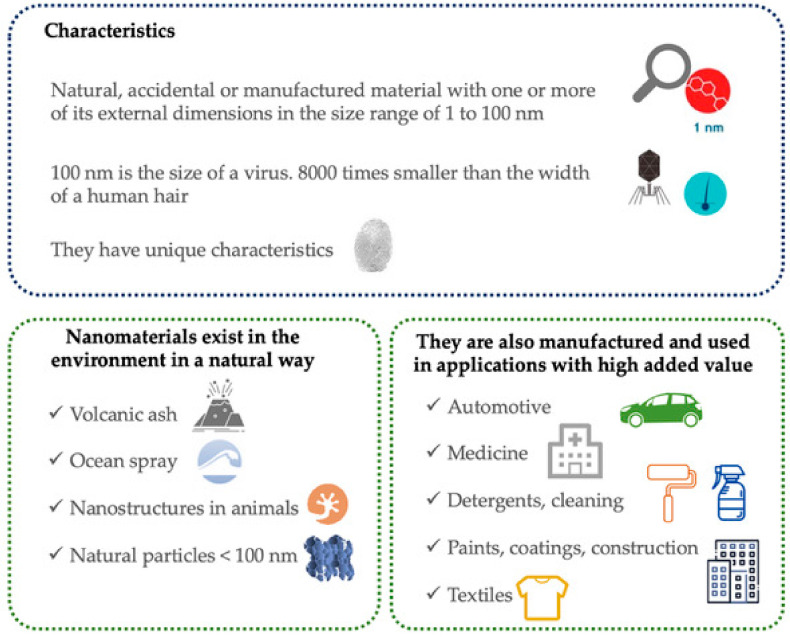
Introduction to nanotechnology, nanomaterials (NMs), and manufactured nanomaterials (MNMs).

Nanotechnology can be defined as a scientific–technical area aimed at the study, development, and use of materials, devices, and functional systems through the control of matter at nanometer scale (10^−9^ m) [1]. Nanomaterials (NMs) that have at least one external dimension in the size range between 1 and 100 nm, can be found naturally in the environment by way of manufacturing, which are commonly called manufactured nanomaterials (MNMs) (see Figure 1).

At this scale, NMMs show peculiar physicochemical properties, such as size, shape, surface area, state of aggregation, or charge, which are different from those of the same materials on the macroscale. Therefore, optical, mechanical, magnetic, electrical, thermal, and biological properties are manifested, for example, properties such as conductivity, solubility, hardness, fire resistance. All these properties allow for unique applications that can have a great impact on different domains, as in, for example, medicine. The construction sector is not unaware of the opportunities involved in the use of these materials and products [1,2,3]. 

Confronted with these benefits, the growth expectations for nanotechnology applications have slowed in recent years due to the uncertainty associated with the potential harmful effects of MNMs on people’s health [4,5,6]. Properties such as their size, reactivity, shape, and solubility are directly related to increased proinflammatory activity and oxidative potential. They may be manifested in the form of respiratory system and cardiovascular diseases and even cancer. The main way of entry to the body is via inhalation, which is especially noteworthy in tasks that involve the generation of powder (cutting, sanding, drilling, etc.) or mists (spray paint) [7]. In Europe, every year more than 1000 workers are killed and over 800,000 workers are injured; others suffer ill-health, such as musculoskeletal disorders, dermatitis, or asbestosis [8]. In a recent international project “Costs and benefits of occupational safety and health,” the researchers estimated that work-related accidents and diseases cost the EU at least 476 billion EUR every year [8]. 

Construction is one of the most dangerous industries due to its own characteristics; for example, it requires a lot of physical work [9,10]. Although the use of MNMs is still somewhat limited, an exponential increase in its application is expected; it has been estimated that 50% of the materials used in 2025 will be nanostructured [11].

Despite the growth that the European nanomaterials market has generated—expected to exceed 9 billion U.S. dollars revenue in 2022 [12]—there is no specific regulatory framework that establishes specific provisions for the evaluation, communication, and risk management for the current uncertainty about toxicological effects and potential exposure.

Regarding workplace safety, occupational exposure limits (OEL) for chemical substances have long been in use for controlling workplace exposures. International Organization for Standardization (ISO) defines OELs as a maximum concentration of airborne contaminants deemed to be acceptable, as defined by the authority having jurisdiction [13]. However, the available studies currently are not sufficient for the establishment of exposure limit values for MNMs. ISO/TR 18637:2016 [14] provides an overview of available methods and procedures for the development of occupational exposure limits and occupational exposure bands, but it does not establish limit values. The challenge is to find consensus on how to derive the OELs for nanomaterials, and next to underpin the proposed values with more empirical research. When there are large deficiencies in hazard data, the National Institute for Occupational Safety and Health of USA NIOSH cites the use of qualitative control-banding methodologies for which several suggestions have been made as an alternative for the Occupational Exposure Limits (OEL)/Recommended Exposure Limit (REL) approach for MNMs. This is also in line with the preferred approach in the UK, where the British Standard Institute (BSI), as a forerunner of the Nano Reference Values (NRVs), developed the idea of guidance values for nanomaterials derived from existing OELs for coarse materials.

Closely linked to the foregoing, it has been demonstrated that they can affect the organism, therefore exposure to NMs can be considered one of the most important new emerging workplace risks (European Agency for Safety and Health at Work [15]), particularly in construction [7]. In this regard, it should be noted also that the European construction sector includes approximately 15 million workers according with the European Federation of Building and Woodworkers (EFBWW), which means a substantial number of workers that can be potentially exposed to hazardous MNMs. In addition, the vast majority of available studies reporting data on workplace measurements between 2000 and 2015 focused on exposure situations in research laboratories and pilot-scale units, resulting in scarce information on the potential exposure during the downstream use of engineered nanomaterials (ENMs) during the professional use of MNMs and nanoenabled products, as is the case of the building sector production.

To overcome this situation in the European Union, the coordinated work of the technical committees working on standardization activities of nanotechnologies and MNMs has allowed the incorporation of modifications in the annexes of the REACH regulation [16]. The working group of the Organization for Economic Cooperation and Development on MNMs is formed by the Working Party Manufactured Nanomaterials (WPMN) and the Competent Authorities for REACH and Classification and Labelling (CARACAL) sub-group for nanomaterials. They are the working group of competent authorities for the implementation of European Regulations related to Registration, Evaluation, Authorization and Restriction of Chemicals (REACH) and on Classification, Labeling and Packaging of substances and mixtures (CLP). In addition, specific information requirements have been defined to ensure their safe use, the review of the Organization for Economic Co-operation and Development (OECD) guidelines, and test methods for the toxicological characterization [17], as well as the definition of strategies, techniques, and sampling protocols for the determination of the concentration of MNMs in the workplace. This represents a first framework of reference for carrying out preventive activity.

Nevertheless, despite current efforts in the field of nanosafety, there are no regulated limit values and international agreements for MNMs, so there are no harmonized references to compare [18]. Meanwhile, the values of non-nano chemical agents should not be used, since the hazard characteristics can be different, given the differences in particle quantity, number, surface area, mass concentration, size distribution, shape, composition, and chemical reactivity [19].

The main objective of this article is to analyze available nanomaterial-related products included in on-line inventories recognized and incorporated in different databases, as well as published inventories. From these products, the safety data sheets (SDS) of construction products based on nanotechnology are analyzed in depth. Moreover, a review of the recommendations on the use of MNMs on site has also been performed. Finally, a protocol is proposed with the aim of serving as a tool for safety technicians on construction sites for the management of MNMs.

## 2. Nanomaterials Background

### 2.1. Typology of NMs

Concerning the definition of nanomaterials, different classifications can be found. For example, the European Commission [20] establishes three groups of NMs: nanopowders and nanostructured powders, nanosuspension, and nanoaerosol. The first of these are individual particles, usually less than 1 mm. The second type describes solid nano-objects dispersed in liquid, while the third are solid nano-objects dispersed in gas, which move freely.

On the other hand, according to the ISO definition [21], a compendium can be compiled of the types of classification that complete and include the groups in the aforementioned EU definition: nanocapsules (particles with an internal structure manufactured at nanometric scale), nanocomposites (consisting of at least one material or two with different phases in which one at least has nanoscale characteristics), nanoemulsion (liquid nano-objects suspended in another liquid), nanoporous (solid materials that contain a small fraction of pores at the nanoscale), and liquid nanospatoms (nanoscale gas bubbles in a liquid). Clearly, MNMs can be presented in suspension, in solid state and with freedom of movement, in the solid state, and fixed or embedded in a solid matrix or surface [22].

In addition, the European Commission after the second review of the regulatory aspects of nanotechnology [21] highlighted some MNMs related to its types and its uses, as well as its applications (Table 1).

Finally, other MNMs that should also be taken into consideration, although with lesser repercussions related to the number of workers exposed to them, are barium sulfate, strontium titanate, strontium carbonate, indium tin oxide, platinum, platinum and palladium alloy, copper nanopowders, nickel nanoparticles, cobalt, manganese, molybdenum, tungsten, lanthanum, lithium, aluminum nitride, silicon nitride, titanium nitride, titanium carbonitride, tungsten carbide, tungsten sulfide, and barium titanate; the latter among the MNMs most used, according to the Jacquet survey [23]. Finally, organic nanoparticles should also be considered, which are cited in the survey conducted by Schmid [24].

### 2.2. Current Status of Regulations

There is a great diversity of regulations and directives that can be applied to users of chemical products throughout the European Union (EU). In addition, several member countries have their own requirements for chemicals that have a national application. In this section, these legislations are briefly described, together with details of how MNMs fit into them.

**Figure 2:**
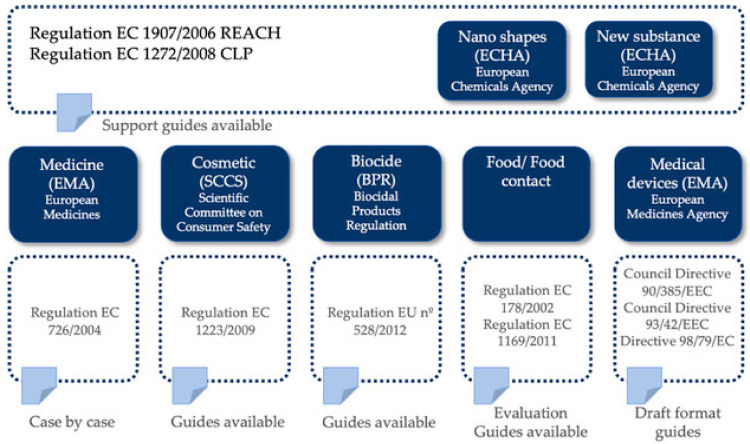
Main regulations applicable to MNMs in Europe.

As mentioned before, in the European legislation there is no specific framework for MNMs, except for regulations relevant to specific sectors that do contain specifications (cosmetics, or materials in contact with food). Currently, the most commonly used regulations for the use of MNMs in the industrial sector are REACH and CLP, both developed by European Chemicals Agency (ECHA) (European Union, 2019). Figure 2 shows the main European Community (EC) and Commission Regulation (EEC) where MNMs are mentioned.

The main current regulatory framework is constituted by Regulation 1907/2006 REACH [25], which aims to ensure that all uses of chemical products that are not covered by a specific regulation are carried out safely within the EU. The regulation requires actions by entities along the whole supply chain of a substance. This covers not only the use of the pure substance, but also the mixtures that contain the substance and the articles manufactured using the substance.

MNMs are generally considered as a form of a substance, rather than a completely different substance. This means that the registration of a substance with nanosized particles is done within the registration dossier of the original substance, together with the bulk forms of the substance. The exemptions to this general rule have been the different allotropes of carbon that have different crystalline structures and forms of particles; for example, diamond graphite, multiwalled carbon nanotubes (MWCNT) and single-walled carbon nanotubes (SWCNT) all have a separate registration dossier.

It should be mentioned that, due to the application of Regulation 1907/2006 for the case of substances of which more than one ton is produced per year (which is still unusual for many MNMs), there are still few results available that contain contrasted information on toxicological properties and exposure levels that ensure the safe use of MNMs in their various forms throughout the supply chain. These include mixtures—for example, construction products—and/or articles, such as polymeric nanostructured materials [25]. Recently, the European Commission published Regulation 2020/878 [26] that amends Annex II to Regulation 1907/2006 and will enter into force on January 21. This regulation updates the SDS for MNMs and introduces specific requirements for nanoforms of substances, as information related to those requirements is to be included in the SDS.

On the other hand, according to the CLP Regulation, MNMs must be classified and labeled. For this, one tool is SDSs, which are designed to provide users of chemical substances with the necessary information to help people and the environment. The format of the SDS is defined in the REACH [27] and is established in 16 sections with different information about the product. This is a key issue in the commercial supply chain, where SDS serves as the most common source of information on whether or not a nanoform of a substance is present. 

By 1 January 2020, companies were required to provide more information on nanomaterials on the EU market under the REACH regulation. ECHA encourages potential registrants to be familiar with the new legal requirements and get ready [16].

In relation to standardization, the Technical Committee (TC) on Nanotechnology of ISO (ISO/TC 229) has not directly addressed the construction sector in ISO/TC 59 Buildings and civil engineering works, ISO/TC 195 Building construction machinery and equipment, or ISO/TC 195 Building construction machinery and equipment. 

In addition to REACH regulation, current Framework Directive 89/391/EEC on the safety and health of workers at work [28] and 98/24/EC on health and safety from the risks related to chemical agents at work also apply to MNMs [29]. 

### 2.3. NMS and Health

#### 2.3.1. Impact on Human Health

In relation to the impact on human health, exposure to nanomaterials has focused on two domains: fabrication and research [30]. In the production and use of NMs, exposure may or may not be related to processes where the purpose is the specific production of NM [31]. In cases where exposure is directly related to the production and use of MNMs, there are three main means of entry: inhalation, dermal, and digestive. Inhalation is the main entry route, being the most worrying regarding occupational health [32,33,34], which is why all studies recommend minimizing exposure to MNM powder, whether it be the manufacturing stage or caused during work processes [33,35]. On the other hand, the dermal route is also relevant in tasks in which a large part of the worker’s body may be in contact with MNMs, since smaller particles can pass through the skin [36]. There are few studies in relation to the dermal route [37]. Finally, the oral route is mainly associated with the involuntary intake of MNMs [38]. 

MNMs in the metabolism follow a general behavior of chemical absorption, distribution, biotransformation, and excretion or elimination. However, NMs have the characteristic of translocation, that is, the property of crossing biological barriers without losing their integrity, and reaching some part of the body inaccessible to non-nanoparticles [39].

#### 2.3.2. Evaluation of Exposure

Despite the lack of knowledge about the levels of MNMs during activities in industrial environments [40], the main reasons limiting evaluation of the exposure potential are the great diversity of MNMs in the market, as well as the lack of consensus on the methodology to detect and quantify these materials in complex environments [41].

In order to evaluate the exposure, most of the works belong to the research domain. Most focus on the characterization of the potential exposure to particles in the nanometer range in synthesis and manipulation processes. 

Different tools have been developed for the hazard assessment methodology, most of them based on control banding (CB) [42]. Appendix A shows different models to assess the exposure to nanoproducts. However, none of them dominates in terms of their applicability or is used more than the others in the different contexts [43]. Liguori et al. [44] checked and compared different tools for CB (the Control Banding Nanotool, IVAM Technical Guidance, Stoffenmanager Nano, ANSES CB Tool, NanoSafer, and the Precautionary Matrix) and showed that, if the inclusion criteria and application domains change, we would need different tools. These are based on different concepts, use different input parameters for evaluation, and obtain results in different formats. Therefore, direct comparison and combinations of the different models is not possible in the short term. In this sense [45] NanoSafer, Stoffenmanager Nano, NanoTool, and the Precautionary Matrix have been evaluated, too. The hazard and exposure classifications were also compared with experimental data. The tools provided different hazard and emission/exposure outputs when compared with each other and with experimental data.

Moreover, the tools are not well enough developed to be used in the construction sector. This is the main goal of this research, to propose an easy tool for use by safety technicians on construction sites for the management of MNMs.

The World Health Organization (WHO) developed the Guidelines on Protecting Workers from Potential Risks of MNMs [46]. This is an important step in protecting workers worldwide from the potential risks of MNMs, and describes five lines to ensure a healthy workplace:

1. Assess health hazards. This recommends assigning a hazard level to MNMs in accordance with the international harmonized labeling system for chemicals (source, ECHA). It also recommends updating of the SDS. However, the specific area in the nanoscale is higher than in the macroscale, so the reactivity will be higher, too, and this hinders the assignation of a hazard level and implies higher safety risks and dangerous atmospheres (explosion and fire) [47,48]. There are studies related to the health effects and the toxicological effects, especially in inhalation, which reveal the higher deposition in the nanoscale, as well as different location (nasopharyngeal, tracheobronchial, alveolar, … [49]. The toxicity depends on the chemical composition of the MNMs, and it increases in the nanoscale. Solubility is another important aspect in the nanoscale, because a soluble material will cease to behave as an MNM [33].
2. Assess potential exposure. It is recommended to use similar methods to those used in industrial hygiene for chemical products, comparing with the limit values (OEL). The problem is that there are no regulated limit values at the nanoscale and the OEL for chemical agents in their macrosize cannot be extrapolated, due to the larger surface area of the MNMs. It is therefore necessary to use other values of recognized prestigious entities, such as the weight values mass of the National Institute for Occupational Safety and Health (NIOSH) or the British Standards Institution (BSI), or the number of particles per volume used by the IFA (Institut für Arbeitsschutz), and the SER (sensitization enhancement ratio) [18,46].
3. Control exposure. Especially in cases with risks of inhalation, since the dermal and digestive routes are less studied, a precautionary approach will be used due to the absence of agreed reference values.
4. Health surveillance. This does not exist, so no specific program is suggested. There are some epidemiological studies for MNMs, with some proposed biomarkers.
5. Training and involvement of workers. At the present moment there are no programs for the training of workers, but they are expected to be developed by 2022.

## 3. Methodology

Due to the absence of recommendations or laws related to the risk management for the use of MNMs in the construction sector and the uncertainty associated with the potential harmful effects of MNMs on worker’s health, a methodology is conducted that is based on discovering and compiling data, and then comparing it with different protocols related to decision-making in construction sector risk management. 

The present work was developed in three stages. 

In the first stage, a search was carried out for formulated products mainly used in construction with MNMs that are incorporated in recognized on-line inventories. This search included the eLCOSH product database, updated by the Center for Research and Training in Construction [50], the base for Statnano on-line data, edited with the support of the Council of Nanotechnology Initiatives of Iran (Nanotechnology Products Database) [51], the Consumer Products Inventory, developed in the framework of the project on emerging nanotechnologies [52], and the Nanodatabase, developed by the Danish Council of Ecology and the Danish Council of Consumer Products [53]. Similarly, the inventories published by the European Association for the Coordination of Consumer Representation in Standardization Activities [54] and the organization “Friends of the Environment and Nature Conservation Association of Germany” were also analyzed (Bund Nanoproduktdatenbank) [55].

Once the recognized MNMs are known, in a second stage the SDSs of products used in construction, included in the last databases and based on nanotechnology, were analyzed in depth. In this analysis, the data include (in the following paragraphs): risk phrases (H phrases), indication of the presence of MNMs in the list of ingredients, specific considerations for MNMs in the exposure section, and protection measures. In this sense, the information in Section 2 (hazards identification), 3 (composition/information on components), 7 (handling and storage), and 8 (exposure controls/personal protection) of each SDS, was organized for best understanding [56], including the forecasted potential exposure to engineered nanomaterials (ENMs) of nanoenabled construction products, according to their use.

In the third stage, an analysis of the recommendations and procedures on the use of MNMs at the construction site was carried out. For this purpose, a search and comparison of the manuals and guides for the management of risks in the construction sector published by different countries of the EU was performed.

**Figure 3:**
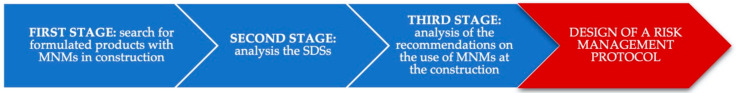
Methodology stage.

Finally, considering the results obtained in the three previous stages, and taking into account the hierarchy control method for risk control, a protocol is proposed. The protocol objective is for it to serve as a tool for technicians responsible for the decision-making management on construction sites regarding the management of MNMs (see Figure 3).

## 4. Results and Discussion

### 4.1. Nanoproducts and MNMs

In the construction sector, the product range also represents the majority of additive products with existing MNMs, as shown by the inventories of nanoproducts that collect the different products available in the market [33,57,58]. Generally, the main applications of MNMs are found in coatings, pigments, and paints [59], in addition to a huge diversity of materials such as ceramics, metals, wood, and stone.

In relation to the types of MNMs used in the construction sector, titanium dioxide, amorphous silica, zinc oxide, and silver are the most prominent [60]. In addition to these, polymeric nanoparticles and aluminum [61] and carbon nanotubes (CNTs) [57,62] are also widely applied.

Another important application in the sector is found in products with photocatalytic activity, capable of self-cleaning surfaces and reducing air pollution or antimicrobial activity [1]. A clear example is found in photocatalytic additive concrete with titanium dioxide (TiO_2_) nanoparticles with antibacterial, self-cleaning, and self-polluting properties that, in addition, lengthen its useful life, helping to maintain its appearance.

On the other hand, there are other important applications related to the improvement of the durability of the materials such as in the case of concrete [63], and the thermal and acoustic insulation properties of glass that are improved with addition of nanosilica gel, while avoiding shadows and annoying reflections. In the case of nanostructured steels, resistances to up to five times greater than with traditional solutions have been achieved.

In painting activities, there is great variety, highlighting anti-graffiti, water- and oil-resistant coatings that prevent other paints from sticking and facilitate subsequent cleaning. Additionally, the MNMs allow for the development of “intelligent materials,” such as construction materials containing nanosensors and nanoparticle self-repair materials.

Table 2 shows a nonexhaustive list of the main ones used in the construction sector, as well as notable properties and types of MNMs included in the formulation and/or matrix of the product.

### 4.2. Management and Recommendations for the Use of MNMs on Site

As mentioned before, there are no specific preventive regulations for working with nanomaterials at the EU level. However, different countries have published specific documents, although they are not binding or mandatory for the construction sector.

In the United Kingdom, the Institution of Occupational Safety and Health (IOSH) published two specific documents for the construction sector: “Nanotechnology in construction and demolition—Guidance for industry” [64] and “Nanotechnology in construction and demolition: what we know, what we do not” [35]. In both of these documents, we find detailed information on the current applications of MNMs in the construction sector. However, they do not provide information on toxicological aspects or the probability of exposure.

Additionally, the German Institute for Work Safety (BAuA) has an interactive website on this subject [65]. Finally, in general, the European Union published the document “Working Safely with Manufactured Nanomaterials” [66], which includes recommendations for use of nanomaterials in activities framed in the construction sector.

In Spain, the National Institute of Safety and Health at Work (INSST) developed different technical tools that aim to facilitate the application of legal requirements. In 2015, the INSST published a document entitled “Risks derived from exposure to MNMs in different sectors: construction” that was updated in 2017 [33]. This document provides information on associated risk, preventive measures, and information on exposure levels in relevant processes in the construction sector.

Regarding the recommendations for use, the possible forms of the most common manufactured nano-objects (MNO) in the construction sector should be taken into consideration. Marcoulaki et al. [67] described diverse forms, and the ones that can be found in this work environment follow:

- Handling and transfer of bulk powdered MNO (e.g., bagging or dumping of powder);
- Dispersion of solid intermediates or ready-to-use MNO-containing products;
- Spraying of ready-to-use nanoproducts;
- Handling of liquid intermediates containing MNOs;
- Activities resulting in fracturing and abrasion of MNO-containing end products (e.g., sanding of surfaces).

In any case, it is necessary to consider the critical factors affecting exposure to MNMs that have been defined by Larrazaza et al. [68]. These factors include the degree of containment, the duration of use, the ability of the material to disperse (in the case of a powder) or form aerosols or drops in the air (in the case of suspensions), and the quality of the material used.

Finally, the precautionary principle recommended by United Nations Educational, Scientific and Cultural Organization (UNESCO) [69] should be implemented, which states: “When human activities may lead to morally unacceptable harm that is scientifically plausible but uncertain, actions shall be taken to avoid or diminish that harm.” 

### 4.3. Protocol for Decision-Making in the Incorporation of MNMs in Construction Work: Implementing Measures for the Prevention of Risks from Their Reception at the Worksite until Waste Management

Prevention of occupational risks is an employer’s responsibility according to Directive 89/391/EEC [21]. Thus, it should be helpful to develop a tool for the task of decision-making in risk management related to the use of MNMs, for implementation by construction sector managers.

Current methodologies for conducting exposure assessment in the workplace are based on a phased approach, starting from a qualitative analysis to the combination of sampling systems and direct reading of instruments in high-precision time.

In the context of the assessment of exposure to MNMs, the main problem with sampling methodologies is their complexity, in addition to not always being representative, since the sampling conditions vary constantly. Another problem to take into consideration is that the measurements involve an additional economic cost, and that the MNMs do not have regulated limit values, so there are no harmonized references with which to make comparisons [18]. In addition to the aforementioned, the effective control measures for exposure to traditional particles could give unsatisfactory results in the case of nanoscale particles [70].

For these reasons, as mentioned before, SDSs are the information tool that technicians use on-site for control. Nevertheless, these files currently lack most of the information on the presence of MNMs in the formulation of products, as well as information on specific management measures [71]. Although there are recommendations for the specific case of MNMs [27,72,73,74], as well as SDSs for products used in construction that contain MNMs [61,75], they still lack relevant safety information for the case of the components in nanoform.

Given this situation, the application of basic norms that apply the hygiene hierarchy of controls [37,76] are fundamental for work with MNMs:

- Elimination of nanoproducts—only in the case that the specific properties of nanomaterials offset possible new risks. However, this option will be a decision considered at the project stage.
- Substitution may be applied with different perspectives in the construction phase, including substitution of nanoproducts, working equipment, and working processes. Among these options is the use of MNMs that generate less dust, the use of liquid matrix additives, or of other equipment that does not generate aerosols, such as the application of paints using rollers instead of sprays [77].
- Engineering controls by means of localized extraction, suction systems, or confinement [33,78].
- Work practices related to both the environment (equipment and work processes—such as placing nonskid mats on the floor so that any material that falls on the mats can be easily cleaned by just removing it [78], or preventing people from moving around a worker handling nanoproducts to avoid air turbulence [79]) and personal hygiene measures (do not store or consume food and drink in the workplace, avoid applying cosmetics, wash hands before eating or leaving the job, and avoid touching your face or other exposed parts of the body with contaminated fingers [80]).
- Personal protective equipment (PPE): the use of gloves, coveralls, respiratory protection, and eye protection [77,81,82].

In addition to the hygiene hierarchy of controls that should be applied, as in any construction activity, the basic principles of preventive action should be taken into account: giving collective protective measures priority over individual protective measures (PPE), as well as providing the necessary information and training of both technicians and construction workers [28]. Finally, the specific health surveillance of personnel exposed to MNMs should not be forgotten [76].

It should be noted that there is currently a trend to apply safe-by-design approaches as a way to mitigate possible risks to human health and the environment. This concept builds on the application of safety measures for the prevention of health damage that are applied during the design stage of a facility, process, material, or product. 

**Figure 4:**
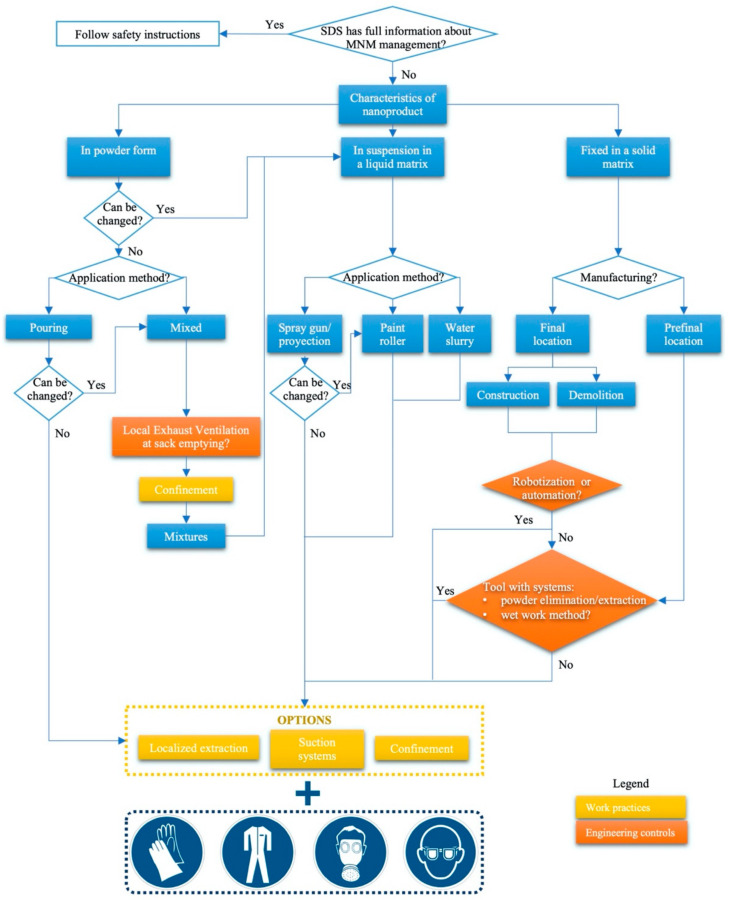
Proposed protocol for decision-making in the incorporation of MNMs in construction work.

As a synthesis of the foregoing, Figure 4 presents the protocol that is proposed as a tool for decision-making in the incorporation of MNMs in construction works. The hygiene hierarchy of controls applicable in the execution phase was taken into consideration. Additionally, for the classification of materials, the one carried out by ISO/TS 12901-2: 2014 [83] is adopted, given that it combines in a simple way the three states in which, mostly, MNMs are received as materials on site:

- Powder-shaped material, as previously discussed, is the most common form that leads to greater exposure.
- Material in suspension in a liquid. This entails direct contact in the manipulation and contact associated with the different forms used on the construction site, such as projection, grouts, cleaning agents (wipes), polishing agents (electric grinding).
- Material dispersed in a solid matrix. This will have different types of machining associated with its assembly: cutting, sanding, drilling, etc. All might produce dust that will contain particles of MNMs.

In the following, some considerations to enable the best understanding and use of the proposed tool are presented:

1. In all issues, before making a decision, the principle of reducing the number of particles released prior to the application of a work practice and the use of PPE have been applied. That is why, if the manufacturer does not provide information on the possibility of changing the method of application of the material in its SDS, the preventive measure will be related to work practices. If a means of application can be selected so as to reduce particle release, this option will be selected.
2. In fact, collective control measures such as ventilation systems are rarely practical on temporary or outdoor sites. Control of airborne dust is usually through a combination of water suppression, maintaining distance (for example, during concrete crushing most workers will be fairly remote from the site of activity, or inside a cab), and the use of personal protective equipment (PPE) [35].
3. The term “final location” refers to those products that are used in the last phase of their life cycle. On the other hand”, prefinal location” refers to those materials that have yet to be handled.
4. It has been considered that, if the robotization/automation option is not possible, tools and/or machinery are used in the wet method. The latter does not exclude the possibility of wetting in these engineering controls.
5. Confinement includes both the limitation of access of personnel to the exposure area and the isolation of the work areas.

Table 3 shows the PPE that are recommended for work with MNMs [37].

## 5. Conclusions

In the last decade, MNMs have experienced an exponential development that is expected to remain unstoppable in the coming years. Their physicochemical properties allow the development of new products with different extraordinary properties, such as optical, mechanical, magnetic, electrical, thermal, and biological.

However, against this potential, MNMs might be the source of adverse effects on the health of workers during their life cycle. Currently, normative regulations have not yet been fully developed and agreed upon.

This means that there are no unified published exposure limits because, among other reasons, they are difficult to establish due to the fact that the levels for which nanoparticles have health effects are unknown.

The construction sector is not unaware of this situation. Technicians working in the execution phase receive materials with NMs and they have available SDSs as a tool for implementing risk prevention measures. However, currently, these files do not provide enough information regarding the presence of MNMs in the formulation of products. Additionally, there is no information concerning specific management measures.

For these reasons, it is necessary to define a noncomplex protocol to serve as a preventive tool that complements the information contained in the SDS. This protocol is based on the application of the precautionary principle defined by UNESCO, incorporating the applicable hygiene hierarchy of controls for the risk management of these MNMs.

The proposed protocol should be an adaptive and flexible tool which feeds off real experiences. The nanospecific risks derived from the use of nanotechnology-based products must be included in the work safety documentation, as well as in the information provided by manufacturers or suppliers in the SDSs. Finally, the coming years are expected to be decisive both at the legislative level and in the search for an international consensus on exposure limit values and the definition of specific preventive measures. This will allow MNMs-based work to stop being considered as an “emerging risk.”

## Figures and Tables

**Table 1:** Manufactured nanomaterials (MNMs) related to their types, uses, and applications.

Inorganic Nonmetallic Nanomaterials	
Synthetic amorphous silica	SiO_2_, EC number 231-545-4
Titanium dioxide	TiO_2_, EC number 236-675-5
Zinc oxide	ZnO, EC number 215-222-5
Aluminum oxide	Al_2_O_3_, EC number 215-691-6
Iron oxides	Fe_2_O_3_, EC number 215-168-2
	Fe_3_O_4_, EC number 215-277-5
Zirconium dioxide	ZrO_2_, EC number 215-227-2
Calcium carbonate	CaCO_3_, EC number 207-439-9
**Metals and Metal Alloys**	
Gold	Au, EC number 231-165-9
Silver	Ag, EC number 231-131-3
**Carbon-based Nanomaterials**	
Fullerenes	
Carbon nanotubes and carbon nanofibers	
Carbon black	EC number 215-609-9
Graphene flakes	

**Table 2:** Nanoproducts and MNMs applied in the construction sector.

Products	MNMs	Properties	Examples
Cement	CNTs	Durability, resistance to cracking, electrical conductivity	EdenCrete (Eden Innovations
	SiO_2_	Mechanical strength, corrosion reduction, reduction of water permeability	Agilia, Ductal, Chronolia (Lafarge) Evolution, Microtech, Promptis (Cemex) Emaco nanocrete (BASF)
	Fe_2_O_3_	Increase in compression force, resistance to abrasion; anticorrosion	Development in research projects
	TiO_2_	Durability, self-cleaning, photocatalytic activity outdoors, improved hydration	NOxer (Eurovia) TioCem (Hanson) Ti Active (Italcementi)
	Graphene	Increased impermeability, bending and plasticity; electric conductivity	Talga Concrete
Paints	Silver (Ag)	Biocidal activity	Ag Bionika Bioni Roof
	TiO_2_	Resistance, photocatalytic activity, biocidal activity, self-cleaning, maintains transparency, hydrophobic	KNOxOUT TM (Boysen^®^) Healthy environment (Granphenstone)

**Table 3:** Recommended personal protective equipment (PPE) for work with MNMs.

PPE	Type
Gloves	Nitrile gloves are generally recommended, but latex is also used [81]
Coveralls	Nonwoven coverall: Tyvek-type [81]
Respiratory protection	FF P3-type disposable masks have been recommended [81] FF P2-type disposable masks have been recommended [65] In this case, we recommend FF P3-type for greater worker protection
Eye protection	As a minimum, close-fitting safety glasses [82]

## Appendix A

**Table A1:** Models to assess exposure to nanoproducts.

Model	Nano Specific? Y/N	Products/ Scenarios	Approach/Principle	Reference
NanoRiskCat	Y	NMs, consumer products, products for professional end-users.	Qualitative ranking. Tiered approach for screening of indications of exposure and effects of MNMs.	[84]
ANSES tool: French Agency for Food, Environmental and Occupational Health & Safety (ANSES) tool	Y	Solids, liquids, powders, aerosols.	Control-banding tool for specific risks in the case of MNMs. Exposure risk estimated considering process location, scaling, and product state along the life cycle studied.	[85]
Swiss Precautionary Matrix	Y	NMs, consumer products, products for professional end-users.	Estimation of the actual (worst-case) airborne exposure or exposure over the course of 24 h or a workday.	[86]
Control Banding Nanotool 2.0	Y	Any operation involving production and use of manufactured NMs.	Control strategies based on overall risk level (i.e., “severity” and “probability” scores) calculated considering amount handled, dustiness, use frequency, workers exposed, and duration of the task, among others.	[87,88]
Stoffenmanager 8.0	N	Stepwise transfer from the source through various transmission compartments to the worker.	Online system to identify the chemical hazards and control the exposure at workplaces. Inhalation model based on the source–receptor approach. Determinants of exposure include task, local controls, general ventilation, and product characteristics.	[89]
Stoffenmanager Dermal module	N	Dermal exposure assessment.	Model uses an algorithm to produce categorical estimates of exposure and risk (and qualitative assessment) considering 15 dermal exposure modifiers to account for differences in surface contact (SC) and deposition (DEP).	[90]
Stoffenmanager Nano	Y	Exposure from point source or fugitive emission during synthesis, handling and transfer of powders, dispersion, application of ready-to-use products, fracturing and abrasion end-products at work sites.	Qualitatively assess occupational health risks ranging from inhalation exposure to ENMs. Input parameters are substance and activity emission potential, localized control, dispersion, surface contamination factor, personal enclosure, personal protection, duration and frequency of the handling.	[91]
NanoSafer 1.0/1.1	Y	Exposure during process-specific manufacturing and handling of NMs.	Control-banding and risk management tool that enables assessment of the risk level and recommended exposure control associated with production and use of MNMs. Calculations based on emission rates (Ei), dustiness data (Eo), activity energy (hi), general (QFF), and NF-FF (QNF) ventilation rates. Outcomes expressed as risk bands in the context of control-banding/risk management banding.	[90,92]
ECETOCTRA	N	Occupational and consumer inhalation, dermal, and oral exposure.	Inhalation exposure is calculated as either the concentration in room air (mg/m^3^) over a day, resulting from one or more events of product/article application or as the inhalation exposure dose (amount per kg body weight (BW)). Estimates may also be made from existing measurement data for a number of consumer and worker. Algorithm for dermal exposure estimation uses such parameters as: dermal dose (mg/kg/day), product ingredient (g/g), contact area (cm^2^), frequency of use (events/day), thickness of layer (cm), density (g/cm^3^), BW. It does not take into account any duration factor and assumes 100% transfer of substance from the product or article contact layer to the skin instantaneously.	[93]
MEASE	N	Occupational inhalation and dermal exposure.	First tier exposure assessment tool developed for the assessment of occupational inhalation and dermal exposure. Inhalation exposure is calculated considering 3 fugacity classes, i.e., low, medium, or high, based on the physical form, the melting point of the metal, the temperature of the process, the vapor pressure, and the selected process category. Dermal exposure estimates, exposed skin area and total dermal loading are also calculated.	[94,95]
EMKG-Expo tool: Easy-to-use workplace control scheme for hazardous substances (EMKG)	N	Inhalation exposure while handling hazardous substances in the workplace.	Chemical banding approach for filtering low-risk workplace situations. The tool uses volatility or dustiness, amount of substance used, and control strategy as three input parameters.	[95,96]
NF/FF model: The Near-field (NF) and the far-field (FF) model	N	Occupational inhalation exposure in NF and FF zones.	Framework for characterization of exposure to ENMs. NF zone comprises the source and workers and FF zone covers the rest of the room. Critical parameters in the NF/FF model are the size of the NF volume, air exchange between the NF and FF volumes, and how well the emission source potency is characterized (i.e., particles emitted from the process to the NF volume).	[97,98]
Advanced REACH Tool	N	Stepwise transfer from the source through various transmission compartments to the worker.	Algorithms estimate the contribution from NF and FF sources. Exposure from a near-field source (Cnf) is a multiplicative function of substance emission potential (E), activity emission potential (H), (primary) localized control (LC1), secondary localized control (LC2), and dispersion (D). The algorithm for a far-field concentration (Cff) also includes segregation (Seg) and personal enclosure/separation (Sep).	[99]
DREAM	N	Pathways resulting in loading of the skin, i.e., through direct contact, by deposition, or transfer from contaminated surfaces.	Initial assessment of dermal exposure, among others, resulting in a ranking of tasks and subsequent jobs. Potential and actual dermal exposure levels are estimated in DREAM units. The estimation of the potential dermal exposure level is based on the product of the “probability” and “intensity” of each exposure route and corrected for 33 determinants at task level. The DREAM model is “generic”, i.e., not taking into account any properties of substances.	[100]
RISKOFDERM	N	Dermal exposures in industrial and professional settings.	RISKOFDERM clusters activities in six different dermal exposure operation (DEO) units based on measured data and the basic estimate is the potential exposure per minute (for hands and/or remainder of the body). For each DEO, different parameters need to be filled in to calculate the dermal exposure.	[101]
dART	N	Dermal deposition.	dART is an extension of ART, with all the ART determinants related to the estimation of air emission applied to predict potential deposition onto the skin, clothing, and surfaces. The total potential exposure to the substance on the skin is a summation of deposition, direct emission, and transfer, including also the weight fraction, retention on the skin or clothing, and the protection of PPE.	[102]
IEAT (Ingestion Exposure Assessment Tool)	N	Estimates hand/object loading from contextual information and uses this to estimate inadvertent ingestion exposure.		[103]
